# Machine Learning to Predict Prostate Artery Embolization Outcomes

**DOI:** 10.1007/s00270-024-03776-z

**Published:** 2024-06-19

**Authors:** G. Vigneswaran, N. Doshi, D. Maclean, T. Bryant, M. Harris, N. Hacking, K. Farrahi, M. Niranjan, S. Modi

**Affiliations:** 1 Department of Interventional Radiology, University Hospital Southampton, Southampton, UK; 2 Faculty of Medicine, Cancer Sciences, University of Southampton, Southampton, UK; 3 Department of Urology, University Hospital Southampton, Southampton, UK; 4 Department of Electronics and Computer Science, University of Southampton, Southampton, UK

**Keywords:** Artificial intelligence, Prostate, Embolization

## Abstract

**Purpose:**

This study leverages pre-procedural data and machine learning (ML) techniques to predict outcomes at one year following prostate artery embolization (PAE).

**Materials and Methods:**

This retrospective analysis combines data from the UK-ROPE registry and patients that underwent PAE at our institution between 2012 and 2023. Traditional ML approaches, including linear regression, lasso regression, ridge regression, decision trees and random forests, were used with leave-one-out cross-validation to predict international prostate symptom score (IPSS) at baseline and change at 1 year. Predictors included age, prostate volume, Qmax (maximum urinary flow rate), post-void residual volume, Abrams-Griffiths number (urodynamics score) and baseline IPSS (for change at 1 year). We also independently confirmed our findings using a separate dataset. An interactive digital user interface was developed to facilitate real-time outcome prediction.

**Results:**

Complete data were available in 128 patients (66.7 ± 6.9 years). All models predicting IPSS demonstrated reasonable performance, with mean absolute error ranging between 4.9–7.3 for baseline IPSS and 5.2–8.2 for change in IPSS. These numbers represent the differences between the patient-reported and model-predicted IPSS scores. Interestingly, the model error in predicting baseline IPSS (based on objective measures alone) significantly correlated with the change in IPSS at 1-year post-PAE (*R*^2 ^= 0.2, *p *< 0.001), forming the basis for our digital user interface.

**Conclusion:**

This study uses ML methods to predict IPSS improvement at 1 year, integrated into a user-friendly interface for real-time prediction. This tool could be used to counsel patients prior to treatment.

## Introduction

Recent advancements in embolization techniques, combined with an increasing body of supporting data, have led to prostate artery embolization (PAE) emerging as a safe and effective alternative to transurethral resection of the prostate (TURP) for the treatment of benign prostatic hyperplasia (BPH) [1]. Although PAE appears to be effective in most patients, there are a subset of patients that have suboptimal outcomes. This can include patients that have an International Prostate Symptom Score (IPSS) reduction of < 25% or no improvement in quality of life (QoL) score, clinical recurrence of symptoms (5–28% of cases) [2] or technical failure (reported as 2–5%) [3]. Given that no procedure is without risk of complications, this has spurred a growing body of research aimed at assessing the underlying predictors of PAE outcomes [4]. Recognising BPH’s heterogeneous nature, a tailored approach to treatment, emphasising the pivotal role of patient selection in both medical and surgical management, will ensure optimal care [4].

Most studies on PAE outcome predictors focus on singular patient factors such as prostate volume, vascular anatomy or IPSS, rather than using a combination of factors [5–9]. Furthermore, predictions are usually based on binary outcomes (responder, non-responder) rather than predictions of actual IPSS scores for that individual patient. Such detailed prediction could be beneficial for clinical decision-making. For instance, even a modest improvement in IPSS might be considered sufficient reason to opt for PAE over more invasive procedures (e.g. TURP), particularly if the latter poses significant risks to the patient.

In the realm of health care, artificial intelligence (AI) signifies a change in thinking, utilising computer systems with data-driven, decision-making processes. Machine learning (ML), a subset of AI, harnesses structured data and algorithms to decipher patterns and predict clinical outcomes. It benefits from the ease of exploring combinations of variables in large datasets to find patterns that might otherwise be missed with traditional statistical approaches. Its success is evident in various medical domains, such as predicting cancer progression (e.g. breast, prostate and lung cancer) and treatment efficacy [10]. Merging prostate volume and clinicopathological data with AI tools holds promise in forecasting PAE outcomes, refining patient outcomes and facilitating tailored patient consultations.

This pilot study seeks to evaluate the feasibility of leveraging ML to predict PAE outcomes, solely relying on pre-procedural routinely collected data (prostate volume, clinical and urodynamic variables).

## Methods

This study was a retrospective complete-case analysis under the ethical approval of IRAS 326704.

### Study Cohort

A retrospective analysis of the UK-ROPE study (a prospectively collected database of patients from the UK) was conducted [11]. Briefly, this was a national observational database of patients treated with PAE or surgical alternatives collated from 17 centres across the UK from January 2014 to July 2016. The inclusion criteria for this subanalysis were patients that underwent PAE and had complete set of records including age, prostate volume, Qmax (maximum urinary flow rate), post-void residual volume, Abrams-Griffiths number (urodynamics score), baseline IPSS and 1-year post-PAE IPSS. In this study, predictor variables were selected based on the number of full datasets available to maximise data points for use in predictions.

This multicentre dataset was combined with a separately collected dataset encompassing patients that underwent PAE at our single institution between 2012 and 2023. These patients also had the same complete sets of predictor variables.

Some of the data from our institution was randomly selected (utilising the ‘random’ function within python) and kept separate from any training data (Dataset 2). This was used for confirmation of findings and to assess generalisability of our model.

All remaining data were used for model development and validation (Dataset 1).

### Model Development

All data analyses were conducted using Python programming environment. Established machine learning algorithms were implemented within the ‘scikit-learn’ library. These included linear regression, ridge regression, lasso regression, decision tree and random forests.

For the small sample size we have in this dataset, more complex methods such as neural networks were not appropriate as they will overtrain and performance will not generalise to other datasets.

The target variables to be predicted were:

1. Change in IPSS at 1 year (baseline IPSS–1-year IPSS)
2. Baseline IPSS—although all patients completed a baseline IPSS questionnaire, we also aimed to evaluate the model’s performance at predicting their baseline IPSS based only on objective clinical measures (termed ‘model-generated baseline IPSS’)*.* The accuracy of this prediction was assessed by calculating the difference between the model-generated baseline IPSS and the actual observed baseline IPSS. This ‘model-generated error’ was then regressed against the change in IPSS (baseline IPSS–1-year IPSS).

### Validation and Model Performance

Model development and validation were performed only on Dataset 1 (see Table 1). For this analysis, models were trained using a leave-out-one cross-validated approach (LOOCV). In this method, one data point from the Dataset 1 was singled out as a validation data point, whilst the remaining data served as the training set. This was carried out so that every data point in turn was the validation set. This method was used to maximise training data and improve performance given the small sample size. Metrics such as mean squared error (MSE), root mean squared error (RMSE), mean absolute error (MAE) and where appropriate R^2^ were used to account for continuous outcome variables (namely IPSS).

**Table 1:** Variables used in analysis for Dataset 1 (model development and validation), Dataset 2 (independent test set) and combined dataset

Factors	Dataset 1-model development and validation (*n* = 112) mean (std)	Dataset 2-independent test set (*n* = 16) mean (std)	Combined dataset (*n* = 128) mean (std)
Age (years)	66.8 (6.7)	65.6 (8.4)	66.7 (6.9)
Prostate volume on US/TRUS/CT/MRI (cc)	109.8 (60.7)	91.9 (47.6)	107.5 (59.4)
Qmax (ml/s)	8.5 (3.9)	7.9 (2.6)	8.4 (3.7)
Residual volume (mls)	179.0 (147.9)	172.4 (158.6)	178.2 (148.7)
Abrams-Griffiths number	75.0 (37.7)	76.4 (35.6)	75.1 (37.4)
Baseline IPSS	21.9 (5.9)	23.2 (3.9)	22.1 (5.7)
IPSS at 1-year post-PAE	10.6 (6.9)	11.2 (6.7)	10.7 (6.8)
Change in IPSS	11.3 (7.3)	12.0 (6.6)	11.4 (7.2)

*US*, Ultrasound; *TRUS*, Transrectal ultrasound; *CT*, Computed tomography; *MRI*, Magnetic resonance imaging; *Qmax*, maximum urinary flow rate; *IPSS*, International Prostate Symptom Score; *PAE*, Prostate artery embolization;

In addition to LOOCV, a further assessment of performance was made on an independent separate dataset (Dataset 2, *n* = 16). This was not used in any model training.

### User Interface Design

‘RShiny’ Dashboard is an ‘R’ based package (available at https://www.rstudio.com/products/shiny/) that has previously been utilised to allow clinician friendly use of computer-based healthcare tools [12, 13]. This package was used to create a custom user interface that incorporated the final model for research purposes and would be subject to regulatory approval prior to routine clinical use.

## Results

### Cohort Demographics

A total of 128 patients were identified. Data from the UK-ROPE study (*n* = 58) were combined with a separate dataset of patients that underwent PAE at our institution (*n* = 70). All predictor variables (age, prostate volume, Qmax, post-void residual volume, Abrams-Griffiths number*,* baseline IPSS and 1-year post-PAE IPSS) were available.

Data were split into Dataset 1 (model development and validation, *n* = 112) and Dataset 2 (independent test set, *n* = 16) as per methods.

Characteristics of each variable are shown in Table 1.

#### Algorithm Performance

##### Change in IPSS

Using Dataset 1, established machine learning algorithms were used to predict the change in IPSS (baseline IPSS–1-year post-PAE IPSS) using a leave-out-one cross-validation approach. Performance metrics are shown in Table 2. These numbers represent the differences between the patient-reported and model-predicted IPSS scores. All models demonstrated modest but similar performance with the mean absolute error ranging between 5.2 for lasso regression model vs 8.2 for the decision tree-based model. Please note that the smaller the error the better the performance.

**Table 2:** Model performance for prediction of change in IPSS (International Prostate Symptom Score) at 1 year using Dataset 1

Model	Mean squared error	Root mean squared error	Mean absolute error
Linear regression	43.96 ± 60.60	5.26 ± 4.04	5.26 ± 4.04
Ridge regression	43.96 ± 60.60	5.26 ± 4.04	5.26 ± 4.04
Lasso regression	43.39 ± 59.86	5.21 ± 4.03	5.21 ± 4.03
Decision tree	95.12 ± 116.09	8.17 ± 5.33	8.17 ± 5.33
Random forests	48.14 ± 61.21	5.69 ± 3.97	5.69 ± 3.97

These numbers represent the differences between the patient-reported and model-predicted Change in IPSS scores

##### Model-Generated Baseline IPSS

Table 3 shows the performance of all models using a leave-out-one cross-validation approach using Dataset 1. Our models performed better across all performance metrics compared with predicting the change in IPSS directly. Again, the best performing model was lasso regression with MAE of 4.94 ± 3.62. Note that the errors of predicting baseline IPSS were smaller than the errors in predicting the change in IPSS (Table 2). This formed the basis for the subsequent analysis below.

**Table 3:** Model performance for prediction of IPSS (International Prostate Symptom Score) at baseline using Dataset 1

Model	Mean squared error	Root mean squared error	Mean absolute error
Linear regression	38.61 ± 52.86	5.00 ± 3.69	5.00 ± 3.69
Ridge regression	38.61 ± 52.85	5.00 ± 3.69	5.00 ± 3.69
Lasso regression	37.53 ± 50.61	4.94 ± 3.62	4.94 ± 3.62
Decision tree	74.83 ± 90.47	7.28 ± 4.68	7.28 ± 4.68
Random forests	39.16 ± 54.81	4.99 ± 3.78	4.99 ± 3.78

These numbers represent the differences between the patient-reported and model-predicted baseline IPSS scores

##### Regression of ‘Model-Generated Baseline IPSS Error’ Against ‘Change in IPSS’

**Fig. 1:**
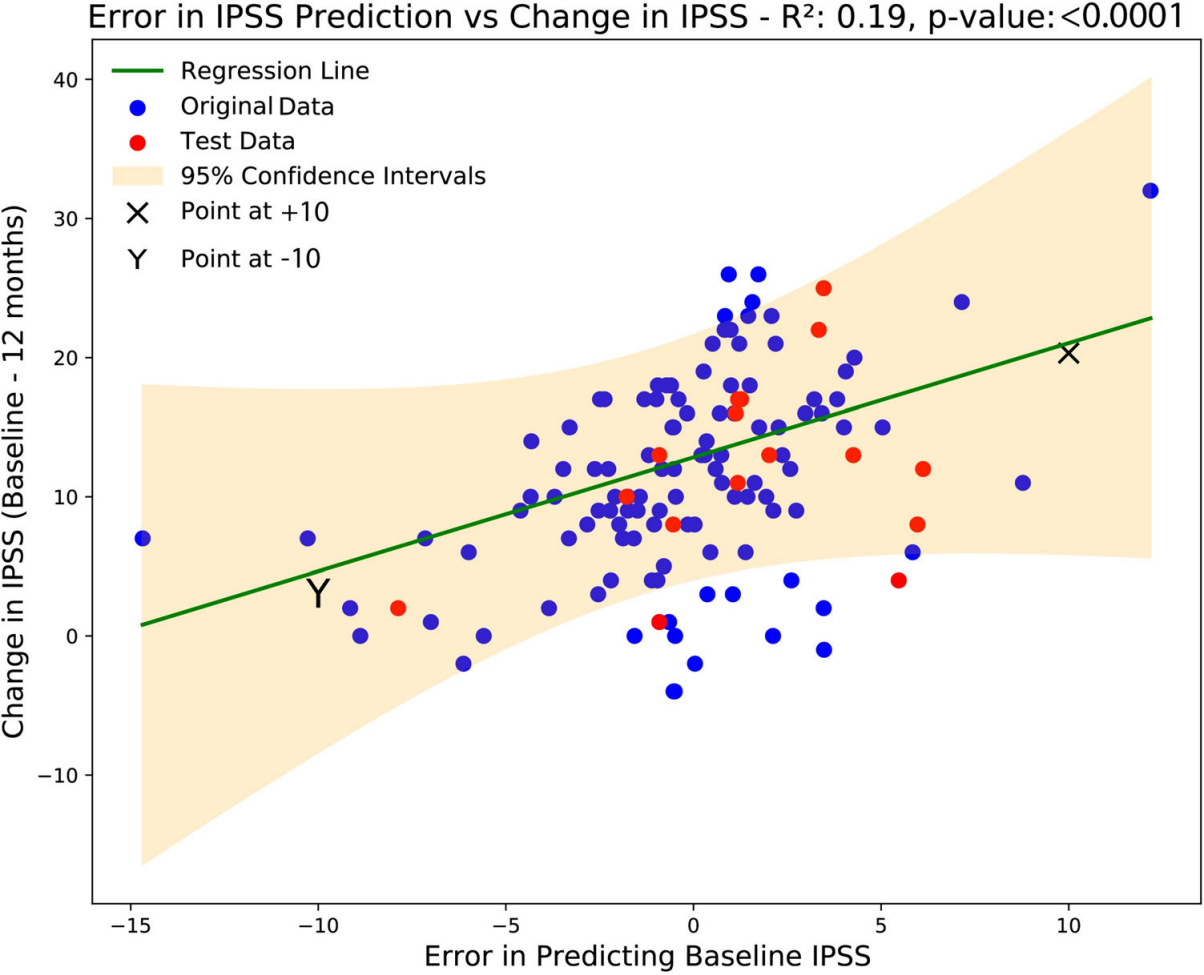
The change in IPSS (baseline – 1-year post-PAE) has a good correlation with the ‘model-generated baseline IPSS error’. Dataset 1 is shown in blue (model development and validation set, *n* = 112) with Dataset 2 superimposed in red (independent test set, *n* = 16). The regression line is plotted in green, and 95% confidence intervals are shaded in orange

To further investigate errors in prediction, the ‘model-generated baseline IPSS error’ was regressed against the change in IPSS at 1 year (baseline IPSS–1-year IPSS). Figure 1 demonstrates that there was a significant relationship between these two variables (R^2^ = 0.2, *p* < 0.001), i.e. patients on whom the model underpredicts IPSS at baseline are those that report the largest improvements in IPSS at 1-year post-procedure. In contrast, patients who reported lower IPSS than predicted had the least benefit. This is further explained below using examples.

##### Examples

1. Mr X had an IPSS of 20 at baseline. Our model estimated from objective measurements including prostate volume, urodynamic and clinical measurements that his ‘model-generated baseline IPSS’ was 10. The error was therefore + 10, and means that he is predicted have a large improvement at 1 year following PAE (**X** on Fig. 1).
2. Mr Y had an IPSS of 15 at baseline. Our ML model estimated that his ‘model-generated baseline IPSS’ is 25. The error was − 10, and this patient was predicted to have little or no benefit of PAE (**Y** in Fig. 1).

### Validation on an Independent Dataset

An independent set of patients who underwent PAE treatment at our institution was used to confirm the generalisability of our findings (Dataset 2). This dataset was held away from the model development process (see methods), and only used in this final stage, Fig. 1 shows that the distribution of these patients (red dots) was similar to Dataset 1 (Blue dots). Indeed, 14/16 (88%) patients were within the 95% confidence intervals of the regression line.

### Towards Implementation

**Fig. 2:**
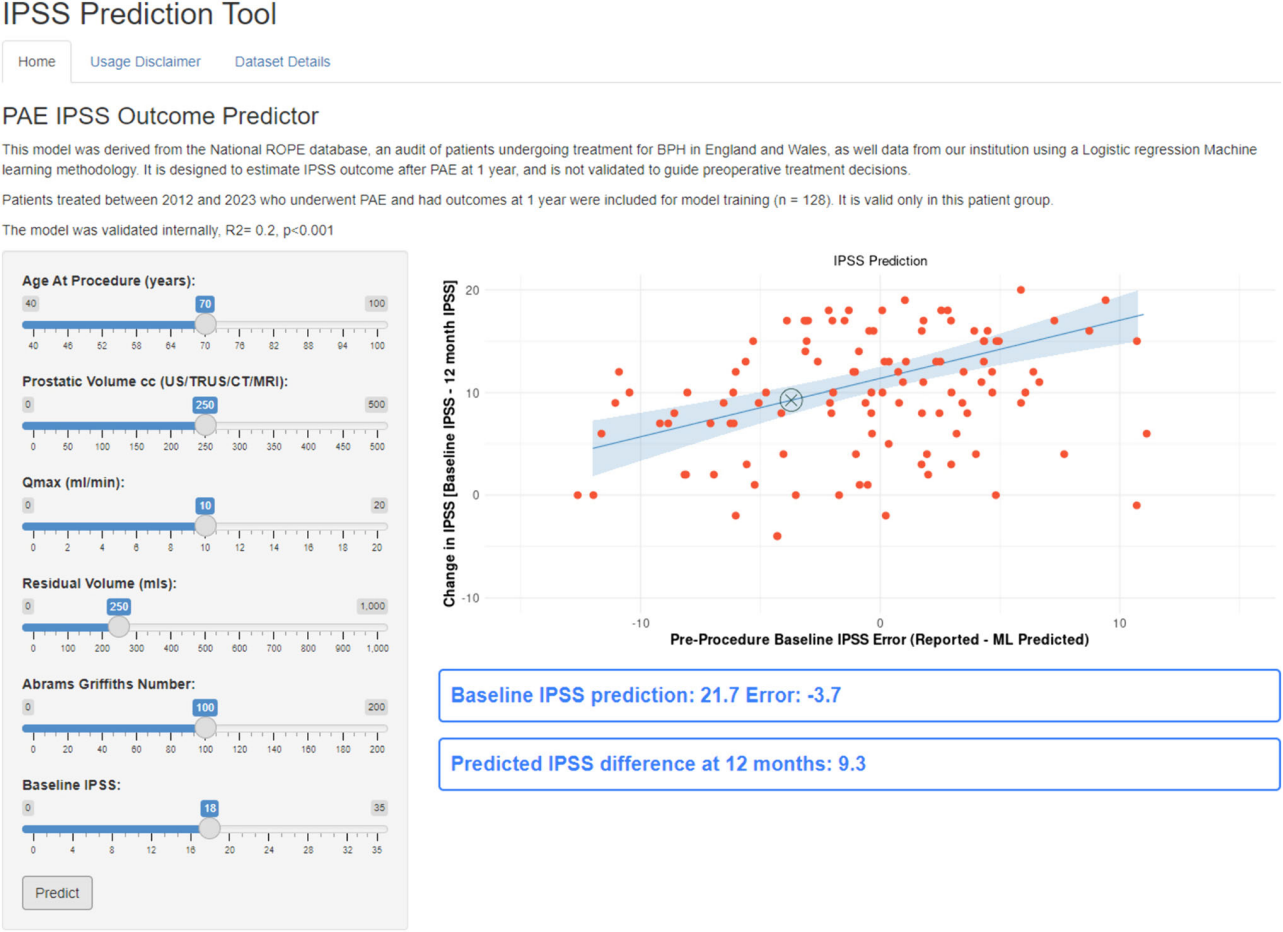
‘RShiny’ dashboard tool screenshot, users are able to adjust individual parameters with sliders on the left side, the prediction of change in IPSS would be displayed as an ‘X’

The final model was incorporated into an interactive digital user interface for illustrative and research purposes (see Fig. 2). This provides an idea of how such a prediction tool might be implemented in a clinical setting for decision support following mandatory regulatory approval. The user can change input predictors with sliders along the left side of the tool and see real-time updates in prediction shown on the graph and right side of the interface.

## Discussion

Our findings suggest that despite using a limited dataset, ML models can be used with routinely collected pre-procedure data to predict the change in IPSS at 1 year following PAE. Interestingly, the most effective way to predict patient outcome was by using purely objective clinical measures to create a ‘model-generated baseline IPSS’. The degree of error between this and the patients’ actual observed IPSS (termed the ‘model-generated baseline IPSS error’) significantly correlated with the ‘change in IPSS’ at 1-year post-procedure and can be used to predict individual patient outcomes with reasonable accuracy.

This finding might reflect a difference between objective and subjective measures of symptoms and points towards a potential psychological element of symptom evaluation through IPSS scoring. Certainly, patient expectations prior to procedures have been shown to significantly influence outcomes. Patients’ beliefs and perceptions about the forthcoming procedure can shape their psychological response, which in turn can influence physiological outcomes, and overall satisfaction with the procedure. For instance, a study by Ellingsen et al. [14] demonstrated that negative expectations could intensify the experience of pain and discomfort. Moreover, when patients hold positive expectations, they are often more compliant with pre- and post-procedure instructions, leading to improved outcomes and decreased complication rates. Indeed, the opposite also applies, in that patients who were adequately informed and thus had clear expectations had shorter recovery times report higher satisfaction rates [15]. This emphasises the importance of effective patient education and setting appropriate expectations to optimise both subjective and objective outcomes in interventional radiology. However, the objective nature of urodynamics is also controversial, e.g. there is some evidence that Qmax is effort dependent and influenced by intervention [16, 17]. Therefore, this is a complex area and needs to be interpreted carefully within this context.

Given the therapeutic intent of PAE is to provide symptomatic relief, it is likely that a combination of psychological and biological factors would lead to symptom improvement. Thus, it remains of pertinent clinical utility to continue using both objective and subjective variables as inputs for any future developed model.

We also found that including a combination of routinely collected variables, notably, prostate volume and urodynamic variables can be used for prediction and is in line with previous studies that have identified prostate volume as a significant predictor of clinical success. Patients with larger prostatic volumes, often above 80 cc, have shown better symptomatic relief post-PAE as compared to those with smaller prostates [18]. This also applied to our model, in which, increasing prostate volume predicts greater IPSS improvement. (We explored this by increasing prostate volume with our tool and observing the predicted change in IPSS rising.) However, critically, it was not this single variable alone that contributed to model performance. Instead our study utilised a combination of factors to predict IPSS outcomes, thereby benefiting from potential performance gains from variable combinations [19]. Machine learning also provides a way in which clinical decision support tools can be improved on subsequent iterations, once additional data are trained, as well as being able to be deployed through interfaces such as ‘Rshiny’ [12].

Notably, our model demonstrated applicability to a separate, blinded dataset (Dataset 2), enhancing the generalisability of our findings. This underscores the potential benefits of establishing a more comprehensive registry of PAE patients. Such an expanded registry could significantly improve model performance, offering deeper insights into patient outcomes and optimising treatment strategies.

Whilst these initial results are promising, it is important to acknowledge the limitations of our study. Firstly, the sample size might mean our models are not representative of the general populations. However, some generalisability was assessed by testing our models on a blinded independent dataset and in part by being trained on multicentre level data. We were also restricted in selecting variables that had full data, as most ML algorithms require complete data. Whilst a single radiological parameter (namely prostate volume) has been used in our models, other radiomic markers might be relevant and imaging data has not been fully utilised. Any future work would also include this readily available and now routinely collected data type, especially given the advantages of performing pre-procedure CT for planning [20]. In addition, clinical measures from formal urodynamic studies were an important component to the ML model. As many centres do not routinely perform urodynamics prior to PAE, this reduces the wider utility of the model and findings.

Furthermore, we emphasise the use of these models as a tool to support clinicians in their decision-making and not to be used as a triaging software independent of clinical oversight.

## Conclusion

This study shows promise in the development of machine learning models that are able to predict individual therapeutic success following PAE from routinely collected clinical data and incorporated into a user-friendly interface. This tool could offer a time critical opportunity for clinical decision-making and patient counselling in Urology and Interventional Radiology Clinics.
